# Supplementary material for: Disentangling listening effort and memory load beyond behavioural evidence: Pupillary response to listening effort during a concurrent memory task
Source: PLoS One. 2021 Mar 3;16(3):e0233251. doi: 10.1371/journal.pone.0233251 (PMC7928507; doi:10.1371/journal.pone.0233251)
Supplement: S1 Appendix — Results and discussions on the alternative method to perform baseline correction using the averaged pupil trace 1s before the first word in the list. (PDF) [file pone.0233251.s001.pdf]

S1 Appendix – Alternative method to calculate PPD

As an alternative to the analysis method presented in the article, baseline pupil diameter in each trial was also calculated as averaged pupil trace 1s before the onset of the first word only, i.e. it was a single baseline for a 10-word list. For clarity, it is referred to here as "block-baseline". The pupil diameter of the rest of the list was subtracted from that block-baseline to obtain relative changes in pupil diameter. This alternative PPD was the maximum diameter of pupil measurements from word onset to response prompt, relative to the block-baseline.

This method has been used sometimes in past literatures, but we did not opt for it because it did not have the power to support the current aim of the study to disentangle the impact of listening effort and memory load on the pupillary response. This being said, we thought it might ease comparisons with other research groups to show the current data with this block-baseline approach, or at least it would highlight its weakness.

Firstly, this alternative PPD yielded one pupillary metric corresponding to the overall cognitive load evoked by word recognition and memory. As depicted in Figure S1a, such a PPD continues to increase from the 1st to the 10th word within the list, when recall is involved. This is a considerable misrepresentation, given that the word-evoked PPD actually *decreases* across words in the list (see Fig. 4c), and the upward trend is due entirely to changes in baseline between words (Fig. 4b) due to memory load accumulation. Similarly, when recall was absent, the alternative PPD exhibits a downward trend from the 1st to the 10th word within the list (Figure S1a), and one is left wondering whether this is due to a reduction in the effort elicited by decoding words or

due to a long-term relaxation of the participant who realizes that the task is much less demanding. Our results (Fig4b, 4c) confirm that both are true but the former effect arises faster than the latter (steeper slope of PPD across word positions than that of baseline).

Looking at the effect of SNR and TASK (Figure S1), the alternative PPD was largely driven by the pattern in pre-word baseline size, due to the smaller range of changes in mm evoked by each word recognition. In repeat-only conditions, the progressive decrease in PPD from quiet to 7 dB contrasts with a sudden jump in PPD for repeat-only at 0 dB. Such a pattern may seem very puzzling, once again because the effect is convoluted between the impact of word-decoding and memory load. Disentangling the two effects let us demonstrate that this is due to a substantial increase in the PPD between 7 and 0 dB SNR (black symbols in Fig 3c), whereas the increase in baseline with acoustic adversity is much more gradual (Fig 3b).

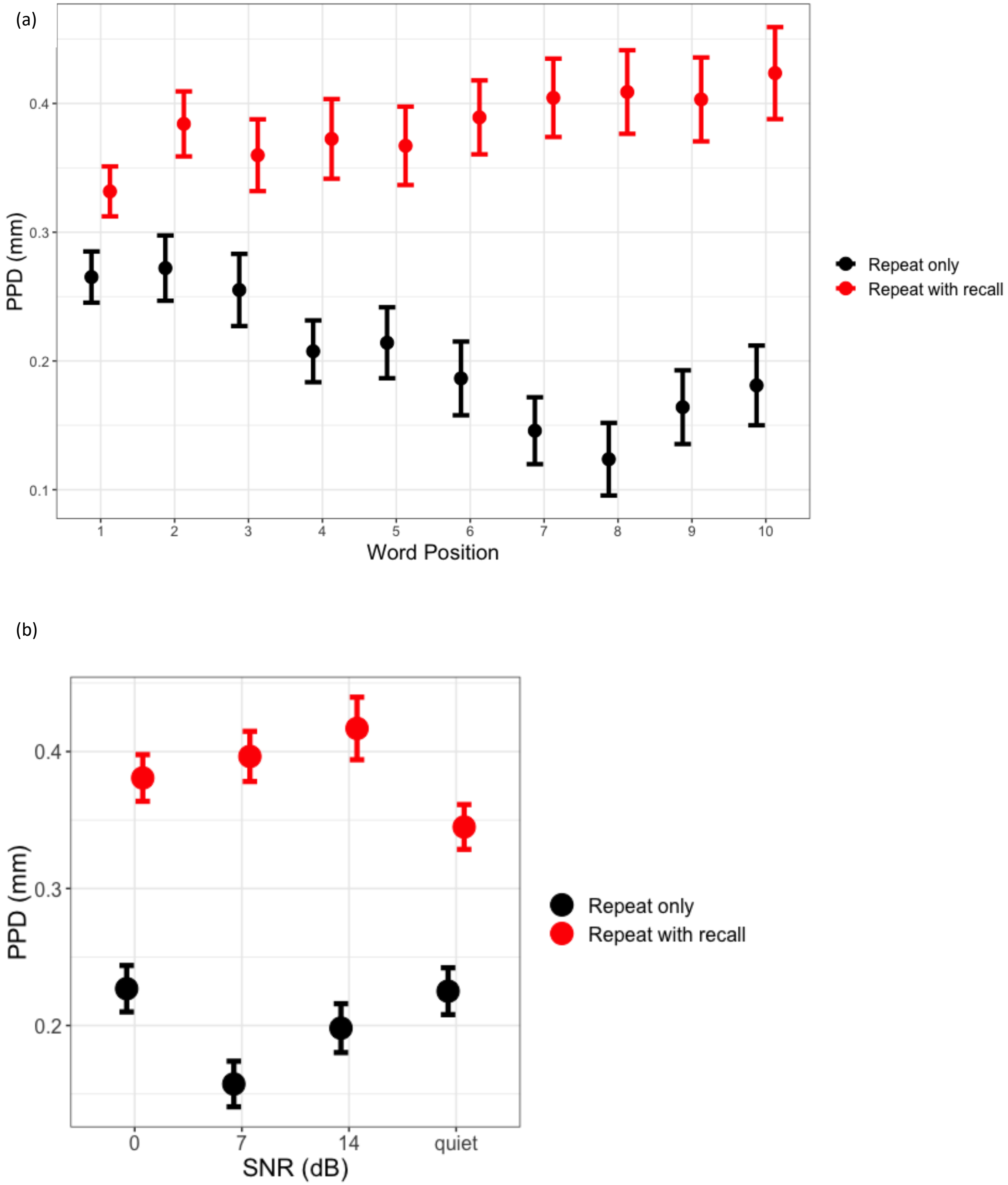

FigureS1. **Alternative pupillometry results calculated using block-baseline.** (a) shows the pupillometry results as a function of TASK and WORD POSITION. (b) shows the results as a function of LISTENING and TASK.
