# Supplementary material for: Disentangling listening effort and memory load beyond behavioural evidence: Pupillary response to listening effort during a concurrent memory task
Source: PLoS One. 2021 Mar 3;16(3):e0233251. doi: 10.1371/journal.pone.0233251 (PMC7928507; doi:10.1371/journal.pone.0233251)
Supplement: S2 Appendix — Model parameter estimates and model comparison statistics for the best fitting models. The reference level for the categorical factor LISTENING is 0dB, for the factor TASK is repeat-only. (PDF) [file pone.0233251.s002.pdf]

S2 Appendix - Model parameter estimates and model comparison statistics.

Model 1: Word recognition performance
Best fitting model: correct ~ SNR * recallOrNot + (1|LISTENER) + (1|WORD LIST)

Fixed effects:	β	SE	p	χ2	df	p
Intercept	0.93	0.11	<0.001			
LISTENING (7dB)	1.96	0.18	<0.001			
LISTENING (14dB)	2.91	0.27	<0.001			
LISTENING (quiet)	4.63	0.58	<0.001	696.88	3	<0.001
TASK (repeat-with-recall)	0.27	0.12	0.03	0.52	1	0.47
LISTENING(7dB):TASK(repeat-with-recall)	-0.27	0.26	0.3			
LISTENING(14dB):TASK(repeat-with-recall)	-0.42	0.36	0.25			
LISTENING(quiet):TASK(repeat-with-recall)	-1.76	0.65	0.007	9.95	3	0.02
**Random effects:**	**SD**			**χ2**	**df**	**p**
Intercept | LISTENER	0.29					
Intercept | WORD LIST	0.42			40.13	1	<0.001
LISTENING | LISTENER				10.25	10	0.42
TASK | LISTENER				0.34	3	0.95
TASK :LISTENING | LISTENER				7.54	45	0.98

Model2: Word recall performance
Best fitting model: nStaCorrect ~ SNR + (1|LISTENER)

Fixed effects:	β	SE	p	χ2	df	p
Intercept	0.32	0.17	0.06			
LISTENING (7dB)	0.38	0.11	<0.001			
LISTENING (14dB)	0.34	0.11	0.003			
LISTENING (quiet)	0.45	0.11	<0.001	18.46	3	<0.001
**Random effects:**	**SD**			**χ2**	**df**	**p**
Intercept | LISTENER	0.77					
Intercept | WORD LIST	0.02			0.1	1	0.99
LISTENING | LISTENER				0.1	3	0.99

Model 3: PPD
Best fitting model: PPD ~ WORD POSITION * TASK + LISTENING*TASK + (1|LISTENER)

Fixed effects:	β	SE	p	χ2	df	p
Intercept	0.29	0.04	<0.001			
LISTENING (7dB)						
LISTENING (14dB)						
LISTENING (quiet)				2.55	3	0.47
WORD POSITION	-0.11	0.04	0.01	104.39	9	<0.001
TASK (repeat-with-recall)	0.01	0.05	0.75	1.85	1	0.17
LISTENING(7dB):TASK(repeat-with-recall)	0.07	0.06	0.29			
LISTENING(14dB):TASK(repeat-with-recall)	0.08	0.06	0.22			
LISTENING(quiet):TASK(repeat-with-recall)	0.07	0.06	0.27	13.15	3	0.004

WORD POSITION : TASK(repeat-with-recall)	0.03	0.06	0.58	22.98	9	0.006
LISTENING : WORD POSITION				20.91	27	0.79
LISTENING : WORD POSITION : TASK				31.05	27	0.27
Random effects:	SD			χ2	df	p
Intercept I LISTENER	0.08					
LISTENING I LISTENER				12.59	9	0.18
TASK I LISTENER				5.93	3	0.11
TASK :LISTENING I LISTENER				12.5	36	0.78

Model 4: Baseline diameter

Best fitting model: Baseline ~ LISTENING + TASK*WORD POSITION + (1ILISTENER)

Fixed effects:	β	SE	p	χ2	df	p
Intercept	3.84	0.16	<0.001			
LISTENING (7dB)	-0.005	0.01	0.67			
LISTENING (14dB)	-0.05	0.01	0.002			
LISTENING (quiet)	-0.03	0.01	0.03	11.21	3	0.01
WORD POSITION	0.06	0.03	0.06	24.85	9	0.003
TASK (repeat-with-recall)	-0.05	0.03	0.98	283.49	1	<0.001
LISTENING(quiet):TASK(repeat-with-recall)				4.11	3	0.25
WORD POSITION : TASK(repeat-with-recall)	0.08	0.05	0.1	82.99	9	<0.001
LISTENING : WORD POSITION				9.41	27	0.99
LISTENING : WORD POSITION : TASK				9.72	27	0.99
Random effects:	SD			χ2	df	p
Intercept I LISTENER	0.7					
LISTENING (7dB) I LISTENER	0.12					
LISTENING (14dB) I LISTENER	0.14					
LISTENING (quiet) I LISTENER	0.14			232.46	10	<0.001
TASK I LISTENER	0.16			245.51	3	<0.001
WORD POSITION I LISTENER	0.08			107.01	55	<0.001

Model 5: Subjective rating

Best fitting model: Rate ~ LISTENING * TASK + (LISTENING:TASK I LISTENER) + (1IWORD LIST)

Fixed effects:	β	SE	p	χ2	df	p
Intercept	-0.15	0.16	0.34			
LISTENING (7dB)	-1.08	0.13	<0.001			
LISTENING (14dB)	-1.34	0.17	<0.001			
LISTENING (quiet)	-1.68	0.19	<0.001	2278.51	3	<0.001
TASK (repeat-with-recall)	1.26	0.25	<0.001	7137.01	1	<0.001
LISTENING(7dB):TASK(repeat-with-recall)	0.26	0.18	0.14			
LISTENING(14dB):TASK(repeat-with-recall)	0.67	0.17	<0.001			
LISTENING(quiet):TASK(repeat-with-recall)	0.59	0.19	0.001	239.78	3	<0.001
Random effects:	SD			χ2	df	p
Intercept I LISTENER	0.39					
Intercept I WORD LIST	0.32			1051.6	1	<0.001
LISTENING (7dB) I LISTENER	0.6					

LISTENING (14dB) ǀ LISTENER	0.8					
LISTENING (quiet) ǀ LISTENER	0.91			1450.35	10	<0.001
TASK ǀ LISTENER	1.21			2210.56	3	<0.001
TASK :LISTENING (7dB) ǀ LISTENER	0.83					
TASK :LISTENING (14dB) ǀ LISTENER	0.78					
TASK :LISTENING (quiet) ǀ LISTENER	0.88			35.69	45	<0.001

Model 6: Pupillary response during recall

Best fitting model: correct ~ LISTENING + (1ǀLISTENER)

Fixed effects:	β	SE	p	χ2	df	p
Intercept	1.29	0.28	<0.001			
LISTENING (7dB)	-0.21	0.38	0.59			
LISTENING (14dB)	-0.31	0.39	0.43			
LISTENING (quiet)	-0.16	0.39	0.68	0.67	3	0.88
Random effects:	SD			χ2	df	p
Intercept ǀ LISTENER	0.32					
LISTENING ǀ LISTENER				0.5	3	0.92
