# Supplementary material for: Disentangling listening effort and memory load beyond behavioural evidence: Pupillary response to listening effort during a concurrent memory task
Source: PLoS One. 2021 Mar 3;16(3):e0233251. doi: 10.1371/journal.pone.0233251 (PMC7928507; doi:10.1371/journal.pone.0233251)
Supplement: S3 Appendix — Analysis on the position of the words recalled in the repeat-with-recall task. (PDF) [file pone.0233251.s003.pdf]

S3 Appendix – Position effect in the word recall task

Besides the overall number of words recalled, the position of the words recalled also provides important information relating to listening effort and memory. The primacy effect is sensitive to listening effort and individual differences [47, 56, 59, 82]. Previous studies have shown that when the speech recognition task is demanding, fewer cognitive resources can be allocated for encoding words from working memory to long-term storage, hence less prominent primacy effect [56]. Listeners with better cognitive capacity have overall more resources to spare so will have more pronounced primacy effect, whereas listeners with worse cognitive capacity are left with resources that can only encode the most recent items [28, 59].

To examine the impact of SNR on the memory encoding over time, the position of the word within a list was analysed. The primacy, asymptote and recency list positions were defined as the 1st to 3rd, 4th to 7th and 8th to 10th words respectively in each word list, similar to that in [47, 57]. The number of words correctly recalled in primacy, asymptote and recency position was counted, and fitted with three logistic mixed-effect models respectively with LISTENING condition as fixed effect factor and LISTENER as random effect factor.

There were significant differences among LISTENING conditions at the asymptotic (χ^2 =8.43, df=3, p=0.04) and recency positions (χ^2 =7.89, df=3, p=0.04), but not at the primacy positions (χ^2 =7.17, df=3, p=0.07).

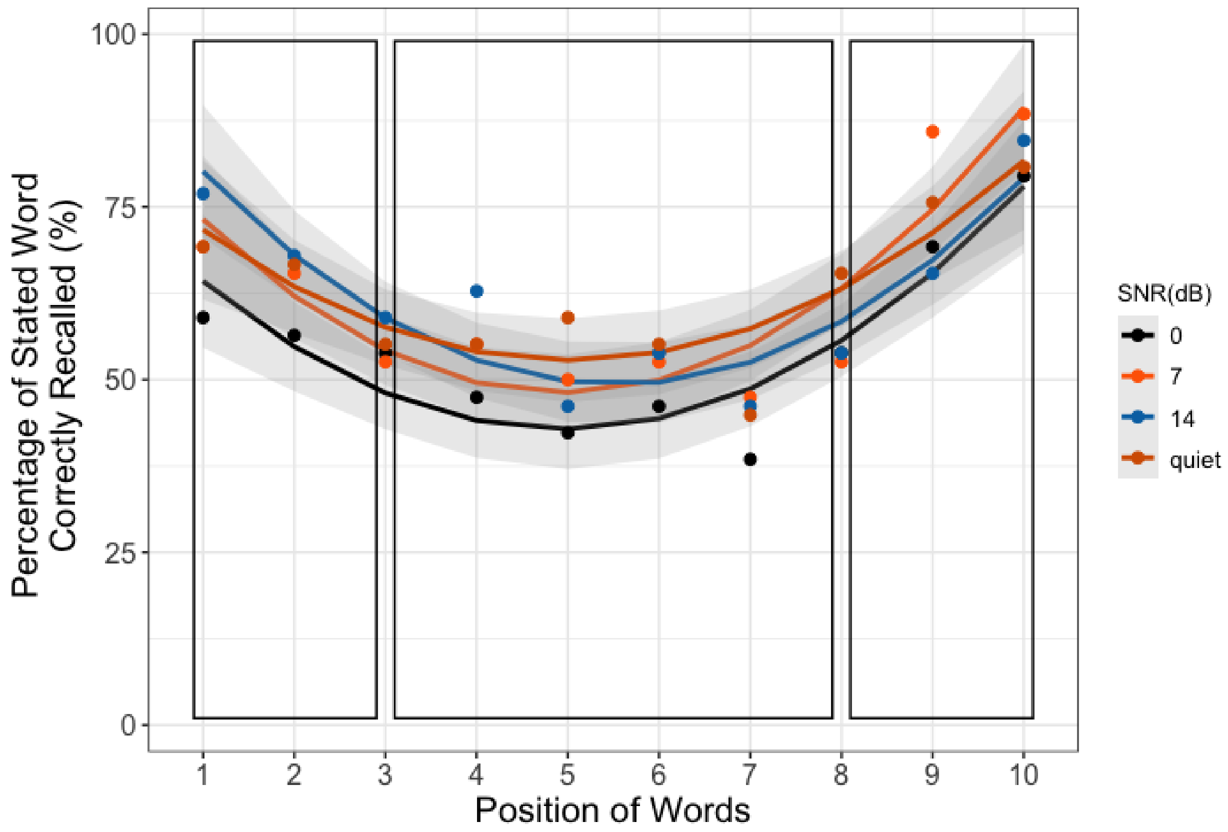

FigureS3. **Position effect in the word recall task in four LISTENING conditions.** All data are averaged across 25 listeners. The shaded width denotes 1 standard error of the mean.
