# Supplementary material for: Disentangling listening effort and memory load beyond behavioural evidence: Pupillary response to listening effort during a concurrent memory task
Source: PLoS One. 2021 Mar 3;16(3):e0233251. doi: 10.1371/journal.pone.0233251 (PMC7928507; doi:10.1371/journal.pone.0233251)
Supplement: Attachment — Submitted filename: Response_to_reviewers_round2.pdf [file pone.0233251.s006.pdf]

Reviewer #1: The revised version of the manuscript gained on clarity and furthermore improved in its readability. I have read the authors' responses and I am happy with most of them. However, two of my major concerns —which are also actually the most important major concerns of my first review— still require authors' response.

I also checked the raw pupil data the authors uploaded. It's great, however, it's unclear if each row represents a time sample. To make the analysis reproducible, could you please also add the time coordinate for each entry?
We have now uploaded pupil data files with extra columns representing aligned sequence in time. They are aligned by the onset of baseline (basAtime), onset of word (wordAtime), onset of word repetition (wordAtime), onset of recall (speakAtime).

REVIEWER 1 IN ROUND 2:
Sorry for being very fussy about this. This has been surprisingly under-addressed in the literature. As you are aware of it now, please properly discuss it in the manuscript and point out that, although it's less exciting, it is a reasonable explanation of your result.

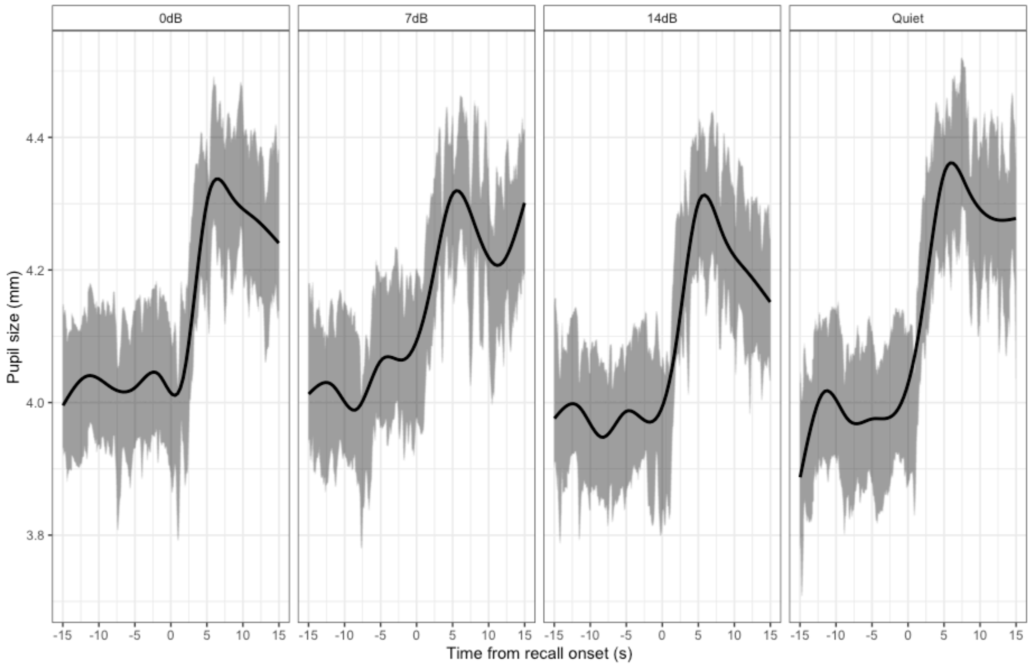

We must not have been clear enough in our first response. It is simply not true that the pupil saturated in the last few words of the lists in the repeat-with-recall condition. And we know that because the pupil jumped by 0.3 mm as soon as the recall started. Please refer to Figure 6 showing the sudden jump which proves that the baseline at the final few trials may be high (around 4 mm), it does NOT prevent the pupil to dilate further. In this regard, we do not need any further experiments to demonstrate that this is case because we have done it: it is shown in Figure 6. Please also find the attached figure to show the pupil trace 15s before the onset of recall. Although every 10-words block varied in time due to different response time, on average 15s covered around the last 5 words in the list. Please notice how, regardless of how high the baseline and PPD already were in the final few seconds during the word listening, pupil size still increased when the recall stage started. It is clear that there is no physical constraints on pupil dilation and the size could increase at the end of the repeat-with-recall block.
(1) I am not convinced by the authors' rejection to regress out the baseline from PPD on a trial basis. As shown in the first paragraph of their response, the authors clearly understood the concern about the correlation between baseline and PPD. As such correlation potentially exist and explains the key result, it should be carefully examined and reported, because it potentially "fully" not just "partially" explains the key finding here. No matter it's a positive or negative correlation, no matter it's within- or across-subjects.
The inherent mathematical relationship between PPD (y-x) and baseline (x) is a theoretical relationship. Even with two variables, x and y, that are completely random and independent of one another, x shares 50% of the variance with x-y. Yet, in practice, there are many factors that can exacerbate this link (i.e.

make its r2 stronger than 0.5) or on the contrary impair this link (i.e. make its r2 weaker than 0.5). But we know that this inherent relationship does NOT fully explain the capping of the pupil towards the end of the list, because as soon as recall starts the pupil (which is at a high baseline, 4 mm) can dilate to a pretty impressive amount, by 0.3 mm. This is twice the PDD observed by the end of the list. Therefore, evidently, the pupil CAN dilate even when the baseline is high. We need no further evidence that the pupil was not mechanically constrained by this baseline being at 4mm.

(2) The authors' response to my second approach is not satisfying either. Remember, the key finding here is the PPD (peak pupil diameter) tends to be smaller in the last few words in the repeat-to-recall condition. The authors need to examine if this is due to the pupillary saturation in the last few words. In other words, the authors need to show that in the last few words, the pupil can still dilate more flexibly just like in other conditions. The dilation from 1st to 10th word (as shown in figure 4b) does not solve the concern at all. Actually, based on figure 4b, the baseline reached a plateau between 4.0-4.1mm after the 6th word, strongly suggesting that the saturation is the case.
We would argue that one of the key finding is also the large jump (which is twice the PPD size) that occurs after the 10th word, while the baseline is at 4 mm. So, figure 4b is not the evidence you are looking for; figure 6 is which demonstrates that there is NO saturation. In other words, the plateau that is visible during the 10 words is not of physical or mathematical limit, but of cognitive origin. This is what we discussed extensively (line 638). And we also cited past study showing similar pupillary response for digit recall tasks (line654).

(3) Moreover, I am not convinced by the authors' statement that "The best interpretation we can offer – and that we discussed – is that listeners would reserve their resources during the 1st to 10th word to retrieve the words during the recall section." This statement is strong, but the link between "small PPD in the last few words" and "reserving the resource" is weak to me. Please elaborate on it.
We are turning around the same point: as the limitation comes from a cognitive source, there must be cognitive reasons why listeners do not dilate more during the listening-and-repeat task, and instead reallocate their resources to the task coming after the 10th word. We elaborated extensively on this topic from line 650 onwards.
——————
MAJOR CONCERN #2

REVIEWER 1 IN ROUND 2:
Thanks.
(1) could you clarify what you meant by "this did not apply well to individual words"? How did you determine that method doesn't apply well?
Very simply, words are short in comparison with sentences, on the order of 700 ms versus 3-4 seconds. As you know, there is intrinsic variability in the pupil dynamics over time. With a longer auditory event (such as a sentence), it is more likely that the pupil will dilate at some point over the next 5 seconds. There may be occasional traces that show a negative peak but they are rare. This is not rare with individual words, as the auditory event is very short and places little demand on the listener (especially here with normally-hearing and relatively young adults). Kuchinsky et al. (2014) used individual words and applied a similar pre-processing method as here, i.e. aggregating across all trials for a given listener and condition. It's not impossible to analyze the data on a trial-by-trial basis but it implies a good deal of variability in the shape of the pupil response, and any naïve reader may feel skeptical to grant meaning to a PPD when the shape of the pupil trace is largely flat or even going down. So, we simply followed recommended practice (Winn et al., 2018).
(2) although it's ok to choose this method as it has been consistently used by Zekveld lab since 2010, it does not mean that in 2020 we, the pupillometry field, should still ONLY reply on this simple method. So, to demonstrate that your result is robust and replicable, please at least report the result with the trial level PPD in supplementary materials.
Of course, not because something has been done in a certain way in the past implies that we should follow it. But here, we very much agree with past recommendations and there are strong reasons why to do so, namely with regard to the stereotypical shape of the pupil trace during an auditory event. In the literature, there are many studies that first average across all trials of a block, and then calculate baseline and PPD. This is NOT what we have done because we believe there is much information to be gained by looking at the evolution of the pupil dynamics within a block (and have shown many pieces of evidence throughout our manuscript to prove it). We calculated baseline and PPD for each position in the block, but since we had three repetitions of each experimental condition, this allowed us to aggregate to a very small degree the three traces of each position, before deriving our pupillary metrics. In contrast to what Review 1 is asking, we have been asked several times through conferences to show the opposite, i.e. first aggregate the traces through all repetitions of a condition (in our case the 30 trials) and derive baseline and PPD from the average traces. We have done so and shown additional analysis

methods in the supplementary materials already. We think that it would be confusing to add yet another analysis method that would differ very little from the present one.
